# Investigating trajectories of social recovery in individuals with first-episode psychosis: a latent class growth analysis

**DOI:** 10.1192/bjp.bp.114.153486

**Published:** 2015-12

**Authors:** Jo Hodgekins, Max Birchwood, Rose Christopher, Max Marshall, Sian Coker, Linda Everard, Helen Lester, Peter Jones, Tim Amos, Swaran Singh, Vimal Sharma, Nick Freemantle, David Fowler

**Affiliations:** **Jo Hodgekins**, BSc, PhD, ClinPsyD, Norwich Medical School, University of East Anglia, Norwich, UK; **Max Birchwood**, PhD, DSc, University of Warwick, Gibbet Hill Road, Coventry, UK; **Rose Christopher**, BSc, Norwich Medical School, University of East Anglia, Norwich, UK; **Max Marshall**, MB BS, MD, University of Manchester, Manchester, UK; **Sian Coker**, BSc, DPhil, Norwich Medical School, University of East Anglia, Norwich, UK; **Linda Everard**, BSc, Birmingham and Solihull NHS Mental Health Foundation Trust, Birmingham, UK; **Helen Lester**, MB, BCH, MD (deceased), previously at the University of Birmingham, Edgbaston, Birmingham, UK; **Peter Jones**, PhD, FMedSci, University of Cambridge, Cambridge, UK; **Tim Amos**, MB BS, MRCPsych, University of Bristol, Bristol, UK; **Swaran Singh**, MBBS, MD, FRCPsych, DM, University of Warwick, Coventry, UK; **Vimal Sharma**, MD, FRCPsych, PhD, University of Chester, Cheshire and Wirral Partnership NHS Foundation Trust; **Nick Freemantle**, MA, PhD, University College London, London; **David Fowler**, MSc, CPsychol, University of Sussex, Brighton, UK

## Abstract

**Background**

Social disability is a hallmark of severe mental illness yet individual differences and factors predicting outcome are largely unknown.

**Aim**

To explore trajectories and predictors of social recovery following a first episode of psychosis (FEP).

**Method**

A sample of 764 individuals with FEP were assessed on entry into early intervention in psychosis (EIP) services and followed up over 12 months. Social recovery profiles were examined using latent class growth analysis.

**Results**

Three types of social recovery profile were identified: Low Stable (66%), Moderate-Increasing (27%), and High-Decreasing (7%). Poor social recovery was predicted by male gender, ethnic minority status, younger age at onset of psychosis, increased negative symptoms, and poor premorbid adjustment.

**Conclusions**

Social disability is prevalent in FEP, although distinct recovery profiles are evident. Where social disability is present on entry into EIP services it can remain stable, highlighting a need for targeted intervention.

Social disability refers to difficulties with social and occupational functioning (i.e. difficulties engaging in meaningful activities and relationships), and has been described as a hallmark of severe mental illness.^1^ There is a large social cost attached to such disability, with a substantial proportion of the estimated cost of psychosis attributed to unemployment and lost productivity (recent estimate £3.4 billion).^2^ As well as a potential consequence of psychosis, it has been suggested that social disability may precede illness onset, with functional impairment also evident in the prodromal phase.^3^

Many aspects of social functioning are affected by psychosis, including employment, relationships and recreational activities. Social and functional outcomes in psychosis are frequently reported to be poor, with follow-up studies suggesting less than 50% of people with a non-affective psychosis achieve a social recovery, with even fewer returning to competitive employment in the long-term.^4^

Evidence suggests that early intervention in psychosis (EIP) may have a positive impact on functional outcome,^5^ but few studies include this area of recovery as a primary outcome and further research is necessary. In addition, problems with measuring social and functional recovery lead to variation in the rates of social disability and recovery reported within the literature.^6^ Measures of social functioning are often confounded with psychotic symptoms, and many were designed for use with individuals with chronic schizophrenia rather than those in the early stages of psychosis. Further research is needed using more valid and accurate assessments of functioning to examine rates and patterns of social recovery over time.

From a research perspective, recovery from first-episode psychosis (FEP) currently tends to be treated as a homogeneous construct, with studies investigating predictors of outcome using group means on measures of functioning, or by comparing FEP samples with non-clinical comparison groups.^7^ Rather than all individuals responding to psychosis in the same way, it is arguably more likely that cohorts of individuals with FEP are heterogeneous, consisting of subgroups with different baseline levels of social disability and different recovery pathways. Identifying different patterns of recovery will be important in developing and implementing targeted recovery-focused interventions. This approach also has face validity in relation to observations of heterogeneity from clinical practice. In the current study, social recovery over a 12-month period following FEP is examined using latent class growth analysis (LCGA) to examine subgroups of individuals with different recovery trajectories. Predictors of these different trajectories are then examined.

## Method

Longitudinal data from the National EDEN study were used for the analyses described in this paper. National EDEN is a national evaluation of EIP services across the UK (services in: Birmingham, Norwich, Cambridge, Cornwall, Bristol and Lancashire), funded by the Department of Health.^8^ The aim of National EDEN was to evaluate the implementation, effectiveness and cost-effectiveness of the first 12 months of care provided by EIP services in the UK. EIP services provide dedicated care to young people aged 14–35 who are experiencing a first episode of psychosis. Further details of care provided by these teams is described in detail in the Policy Implementation Guide.^9^ Consecutive patients accepted into each EIP service from August 2005 to April 2009 were approached and invited to take part in the study. Participants were assessed up to 3 months post acceptance into EIP (baseline), and 6 and 12 months later. A total of 1027 individuals consented to take part, with 80% followed up at 6 months and 77% followed up at 12 months.

### Participants

The sample included in this study are 764 participants (74%) from the National EDEN study who completed the Time Use Survey (TUS) at baseline and at least one other time point (6 months and/or 12 months). Participants who completed the TUS did not differ from participants who did not in terms of age at onset of psychosis, diagnosis, duration of untreated psychosis (DUP), gender, ethnicity and work status. Demographic information about the sample is provided in Table 1.

**Table 1:** Demographic characteristics of sample (*n* = 764)

Characteristic	
Age at onset, years: mean (s.d.)	21.29 (5.03)

Duration of untreated psychosis >4 months, *n* (%)	320 (41.9)

Diagnosis, *n* (%)
Psychosis	595 (77.9)
Schizophrenia	70 (9.2)
Bipolar/schizoaffective disorder	49 (6.4)

Gender, *n* (%)	
Male	532 (69.6)
Female	232 (30.4)

Ethnicity, *n* (%)	
White	556 (72.8)
Asian	116 (15.2)
Black Caribbean	53 (6.9)
Mixed ethnicity	39 (5.1)

Not in education, employment or training, *n* (%)	437 (57.2)

### Measures

#### Time use survey (TUS)^10,11^

Social recovery was assessed using the TUS at baseline, 6 months and 12 months to measure weekly hours engaged in structured activity. The TUS administered in the study was a shortened version of the individual questionnaire originally used by the Office for National Statistics,^10^ in a national survey to examine how members of the population of the UK spend their time.

Categories of activity included in the TUS are: work, education, voluntary work, housework and childcare, leisure, and sports. Lists of activities are provided for each category (e.g. leisure activities include going to the cinema, pub and eating out). Participants are asked how many times they had engaged in each activity over the past month and for how long on each occasion. A weekly average is then calculated for hours spent in structured activity over the past month (paid/voluntary work, education, childcare and chores, and structured social activities). In the current study, the TUS was administered by trained interviewers, taking approximately 20 min to complete (interrater reliability, intraclass correlation = 0.99).

On average, a non-clinical sample aged between 16 and 36 years spends 63.5 h per week in structured activity (data from the Office for National Statistics).^10^ The TUS has been successfully used with a sample of individuals with psychosis enabling clinical cut-off scores to be established.^11^ A cut-off score of 45 h per week is indicative of good social functioning (i.e. within the non-clinical range). Individuals scoring between 30 and 45 h per week can be defined as at-risk of social disability. Individuals scoring below 30 h per week can be defined as experiencing social disability; and individuals scoring below 15 h per week can be defined as experiencing severe social disability.

#### Candidate explanatory variables

Potential explanatory variables were assessed at baseline. DUP was assessed retrospectively using the method described by Larsen *et al*^12^ using notes and participant reports to ascertain the length of time between the onset of psychotic symptoms and the start of criterion treatment. A dichotomous DUP variable was then constructed representing short (<4 months) and long (>4 months) DUP based on suggestions in the current literature that there is likely to be a ‘critical period’ of DUP.^13^ The Premorbid Adjustment Scale (PAS)^14^ was used to assess participants' self-reported functioning prior to the onset of their psychotic episode in childhood (up to age 11), early adolescence (12–15 years), and late adolescence (16–18 years). The Positive and Negative Syndrome Scale (PANSS)^15^ was used to assess the frequency and severity of psychotic symptomatology (positive, negative, and general) and the Calgary Depression Scale (CDS)^16^ was used to assess symptoms of depression.

### Analysis plan

#### Identifying trajectories of social recovery

Latent class growth analysis (LCGA) is a technique developed by Nagin^17^ for identifying distinct homogeneous subpopulations with similar trajectories of growth over time (known as latent classes) within longitudinal data collected from a larger heterogeneous population.^18^ LCGA is a type of growth mixture modelling where the variance and covariance estimates for growth factors within each class are fixed to zero, assuming homogeneity within classes.^19^ The analyses were conducted using Mplus version 4.^20^ As data were only available for three time points, only linear growth curves could be fitted. Models with varying numbers of latent classes were fitted to the data, increasing the number of classes until the model which best fitted the data was identified. The best-fitting model was chosen according to the following fit indices: Bayesian Information Criterion (BIC), Lo-Mendell-Rubin Likelihood Ratio Test (LMR-LRT), and Bootstrapped Likelihood Ratio Test (BLRT). Lower BIC values suggest more parsimonious model fit, whereas a significant LMR-LRT or BLRT value suggests that a K class model fits the data better than a K-1 model, i.e. an additional class improves model fit. Interpretability of the successive models were also considered alongside fit indices.

#### Examining predictors of social recovery.

The estimated recovery trajectory classes were saved and imported into SPSS version 16^21^ for further exploratory analyses. Classes of individuals with different social recovery trajectories were compared on baseline predictor variables using one-way ANOVAs and chi-squared tests. *Post hoc* tests were conducted to interpret significant main effects. Multinomial regression was used to examine predictors of recovery trajectory, with social recovery trajectory as the response variable (reference category = Low Stable trajectory) and candidate explanatory variables including: gender; ethnicity; DUP; age at onset of psychosis; positive, negative, and general psychotic symptoms; depression; and premorbid adjustment. Multinomial regression was used as the recovery trajectories were treated as nominal, rather than ranked ordinal, categorical variables. A Bonferroni corrected *P*-value of *P*<0.004 (0.05/14) was used to establish statistical significance when conducting multiple comparisons, to correct for family-wise error.

## Results

### Descriptive statistics

Descriptive statistics for TUS scores at each of the three time points and predictor variables at baseline are shown in Table 2. The proportion of individuals scoring on each of the specified cut-offs on the TUS at each time point is shown in the online data supplement (Table DS1).

**Table 2:** Descriptive data for all variables

	*n*	Minimum-maximum	Median	Mean (s.d.)
Structured activity, hours per week				
Baseline	764	0–140	15.25	25.07 (26.23)
6 months	673	0–140	24.00	30.82 (25.28)
12 months	623	0–136	26.50	32.49 (26.97)
PANSS positive symptoms	727	7–33	15.00	15.09 (5.87)
PANSS negative symptoms	718	7–43	14.00	15.14 (6.56)
PANSS general symptoms	722	16–79	32.00	32.94 (9.96)
Calgary Depression Scale	736	0–26	5.00	6.37 (5.36)

Premorbid adjustment				
Childhood	715	0–0.88	0.21	0.23 (0.18)
Early adolescence	691	0–0.77	0.27	0.29 (0.17)
Late adolescence	586	0–0.93	0.30	0.31 (0.19)

PANSS, Positive and Negative Syndrome Scale.

### Latent classes of recovery

Growth models with varying numbers of latent classes were fitted to the data, increasing the number of classes until the model which best fitted the data was identified. Table 3 shows the model fit for all LCGA models assessed. Overall, a three-class model described the data well and was a significantly better fit than a model with two classes, according to both the LMR-LRT and the BLRT statistics. Although the BIC value reduced further for models with four and more classes, and these models were deemed a better fit according to the BLRT, a three-class model was chosen for reasons of parsimony and interpretability. Models with four and more classes included classes consisting of less than 5% of the sample and did not add any further interpretive value. This was also supported by a non-significant LMR-LRT value for models with four and more classes.

**Table 3:** Criteria for deciding the number of classes within the repeated measures of time use

Number of classes	Number of free parameters	BIC	LMR-LRT statistic	LMR-LRT *P*	BLRT statistic	BLRT *P*
1	5	18 851.91	–	–	–	–

2	8	18 809.33	508.57	<0.001	534.11	<0.001

**3**	**11**	**18 652.52**	**168.28**	**0.05**	**176.73**	<**0.001**

4	14	18 582.12	86.00	0.32	90.32	<0.001

5	17	18 506.09	91.36	0.10	95.94	<0.001

6	20	18 452.27	70.76	0.27	74.32	<0.001

BIC, Bayesian Information Criterion; LMR-LRT, Lo-Mendell-Rubin Likelihood Ratio Test; BLRT, Bootstrapped Likelihood Ratio Test.

Values in bold represent the model fit for the best-fitting model.

**Fig. 1:**
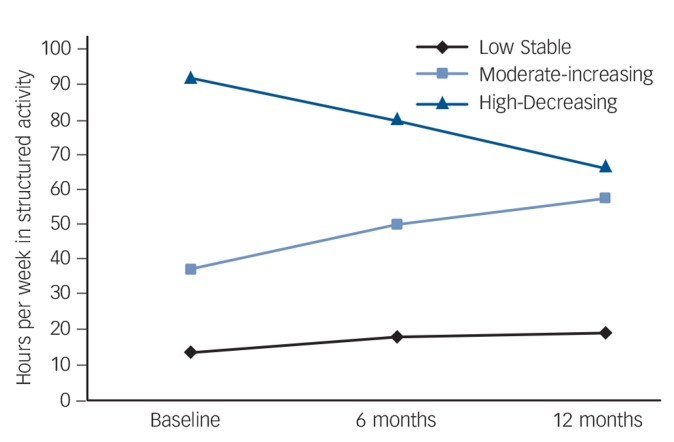
Latent class growth analysis model with three social recovery trajectories.

Average class probabilities for the three-class model were high (0.84–0.94), indicating participants were correctly assigned to their respective latent classes. Convergence checks were conducted on the three-class model to ensure that it was not a local solution.^18^ Model estimates were replicated, suggesting a global solution and increasing the stability of the findings. Thus, a three-class model was chosen as the best fitting model and is illustrated in Fig. 1.

#### Class 1 – low stable time use

The first class contained the largest number of participants (*n* = 507, 66.3% of the sample) and was characterised by a trajectory with a low intercept (unstandardised mean intercept = 14.00) and a shallow but significant positive slope (unstandardised mean slope = 3.18, *P* = 0.001). This class was labelled the Low Stable group, reflecting individuals with high levels of social disability at baseline which remained relatively stable and below the non-clinical range over the study period.

#### Class 2 – moderate-increasing time use

The second class (*n* = 204, 26.7% of the sample) was characterised by a trajectory with a moderate intercept (unstandardised mean intercept = 36.64) and a significant positive slope (unstandardised mean slope = 10.29, *P*<0.001). This class was labelled the Moderate-Increasing group, reflecting individuals with a moderate level of social disability at baseline which improved over the study period into the non-clinical range.

#### Class 3 – high-decreasing time use

The third class (*n* = 53, 6.9% of the sample) was characterised by a trajectory with a high intercept (unstandardised mean intercept = 90.53) and a significant negative slope (unstandardised mean slope = −12.78, *P* = 0.008). This class was labelled the High-Decreasing group, reflecting individuals who were not socially disabled at baseline (scoring above the non-clinical range) and, despite a large decrease, maintained their level of functioning within the non-clinical range throughout the 12-month study period. Nonetheless, there was a significant deterioration in time spent in structured activity in this group which could become problematic if it did not stabilise following the 12-month period.

### Between-group differences

The three classes (Low Stable, Moderate-Increasing and High-Decreasing) were compared on demographic characteristics and baseline predictor variables. Descriptive statistics are shown in Table 4. Group differences were found in age at onset of psychosis, gender, ethnicity, psychotic symptoms and premorbid adjustment. The Low Stable group had a younger age at onset of their psychosis, higher baseline levels of psychotic symptoms (positive, negative and general), and poorer premorbid adjustment in adolescence. There were also a higher proportion of males and individuals from ethnic minority groups in the low stable group. There were no group differences in DUP, baseline depression scores or premorbid adjustment in childhood up to 11 years. After applying a Bonferroni correction for multiple comparisons (*P*<0.004), all between-group differences remained significant.

**Table 4:** Descriptive statistics for baseline predictor variables for social recovery classes

	Low Stable (*n* = 507)	Moderate-Increasing (*n* = 204)	High-Decreasing (*n* = 53)	Statistic
Age at onset of psychosis, years: mean (s.d.)	20.75 (4.67)*	21.88 (5.32)**	24.30 (5.97)***	*F* (2, 728) = 13.58, *P*<0.001

Male, *n* (%)	378 (75)*	128 (63)**	26 (49)**	χ^2^ (2) = 21.00, *P* = <0.001

Ethnicity, *n* (%)				χ^2^ (6) = 21.97, *P*< 0.001
White	346 (68)	169 (83)	41 (77)	
Asian	93 (18)*	17 (8)**	6 (11)**	
Black African-Caribbean	44 (9)*	7 (4)**	2 (4)**	
Mixed ethnicity	24 (5)	11 (5)	4 (8)	

DUP > 4 months, *n* (%)	217 (43)	76 (38)	27 (51)	χ^2^ (2) = 3.64, *P* = 0.16

PANSS positive, mean (s.d.)	15.63 (5.85)*	13.98 (5.93)**	14.35 (5.23)	*F* (2, 724) = 6.04, *P* = 0.002

PANSS negative, mean (s.d.)	16.16 (6.73)*	13.45 (5.95)**	12.00 (4.59)**	*F* (2, 715) = 18.92, *P*=0.001

PANSS general, mean (s.d.)	33.84 (9.91)*	31.28 (10.37)**	30.94 (7.67)	*F* (2, 719) = 5.73, *P* = 0.003

Calgary Depression Scale, mean (s.d.)	6.47 (5.44)	5.91 (5.16)	7.13 (5.31)	*F* (2, 733) = 1.33, *P* = 0.27

Premorbid adjustment, mean (s.d.)				
Childhood	0.24 (0.18)	0.21 (0.18)	0.26 (0.18)	*F* (2, 712) = 2.05, *P* = 0.13
Early adolescence	0.31 (0.17)*	0.25 (0.17)**	0.25 (0.16)	*F* (2, 688) = 9.42, *P*<0.001
Late adolescence	0.35 (0.19)*	0.24 (0.17)**	0.23 (0.15)**	*F* (2, 583) = 21.10, *P*=0.001

DUP, duration of untreated psychosis; PANSS, Positive and Negative Syndrome Scale.

* *P*<0.05

** *P*<0.01

*** *P*<0.001.

Significant differences are between groups on *post hoc* tests.

### Predictors of recovery

Baseline predictor variables were entered into a multinomial regression model with recovery latent class as the dependent variable, using the Low Stable recovery trajectory as the reference category. The results of the regression model are shown in Table 5.

**Table 5:** Results of multinomial regression analysis for social recovery trajectory

	*B*	*s.e.*	Odds ratio
*Moderate-Increasing v. Low Stable*			
Intercept	0.39	0.67	
Age at onset of psychosis	0.33	0.24	1.03
Positive symptoms	−0.06	0.03	0.95*
Negative symptoms	−0.03	0.02	0.97
General symptoms	−0.01	0.02	1.00
Depression	−0.01	0.03	0.99
Premorbid adjustment (up to 11 years)	0.96	0.87	2.61
Premorbid adjustment (12–15 years)	−0.16	1.07	0.86
Premorbid adjustment (16–18 years)	−2.64	0.91	0.07**
Gender (females *v.* males)	0.48	0.24	1.61*
Ethnicity (base = White British)			
Asian	−1.06	0.38	0.35**
Black African–Caribbean	−1.74	0.58	0.18**
Mixed ethnicity	0.23	0.46	1.26
Duration untreated psychosis (short *v*. long)	0.25	0.23	1.28

*High-Decreasing v. Low Stable*			
Intercept	−2.63	1.10	
Age at onset of psychosis	0.12	0.04	1.12***
Positive symptoms	−0.04	0.05	0.96
Negative symptoms	−0.10	0.05	0.91*
General symptoms	0.02	0.04	1.02
Depression	0.01	0.04	1.01
Premorbid adjustment (up to 11 years)	2.83	1.44	17.00*
Premorbid adjustment (12–15 years)	−1.35	1.95	0.26
Premorbid adjustment (16–18 years)	−3.88	1.78	0.02*
Gender (males *v.* females)	0.77	0.39	2.16*
Ethnicity (base = White British)			
Asian	0.05	0.56	1.05
Black African–Caribbean	−0.38	0.83	0.68
Mixed ethnicity	0.37	0.84	1.45
Duration untreated psychosis (short *v.* long)	−0.44	0.39	0.64

Nagelkerke pseudo *R*^2^ = 22.9%. Model χ^2^ = 103.80, *P*<0.001.

* *P* <0.05

** *P* <0.01

*** *P* <0.001.

Compared with individuals with a low stable trajectory, individuals with a moderate-increasing trajectory were more likely to be female, less likely to have ethnic minority status, have lower levels of positive symptoms at baseline, and better premorbid adjustment (lower scores on the PAS) in late adolescence.

Compared with individuals with a low stable trajectory, individuals with a high-decreasing trajectory were also more likely to be female and to have better premorbid adjustment in late adolescence. In addition, they were more likely to have lower baseline negative symptoms and an older age at onset of psychosis. Interestingly, individuals with a high-decreasing trajectory were also more likely to have poorer premorbid adjustment (higher scores on the PAS) in childhood, compared with those with a low stable trajectory.

The results suggest that being male and having an ethnic minority status may be associated with a poorer social recovery trajectory. Moreover, high baseline levels of negative symptoms, poor premorbid adjustment in adolescence, and a younger age at onset of psychosis may also increase the likelihood of a poor functional outcome.

## Discussion

### Summary of results

This study utilised LCGA to examine trajectories of social functioning in a large longitudinal dataset. The results suggest that social recovery from FEP is heterogeneous. A large proportion of individuals displayed a high level of social disability which did not improve over the first 12 months of service provision (66%). However, there was also a minority who did not display social disability at baseline or follow-up, scoring above the non-clinical cut-off on the TUS, but who nevertheless experienced a significant reduction in time use over the 12-month period (7%). A further group experienced moderate levels of social disability when presenting with their first episode of psychosis but demonstrated an improvement in functioning over the 12-month period (27%). Factors predicting poor social recovery over time included male gender, poor adolescent premorbid adjustment, high baseline levels of negative symptoms, ethnic minority status, and a younger age at onset of psychosis.

### Interpretation of findings

#### Rates of recovery

Participants in the Moderate-Increasing group (27%) had a good outcome, with their functioning improving over time and reaching the non-clinical range at the end of the 12-month period. It could be argued that participants in the High-Decreasing group (7%) also had a good outcome as their functioning remained in the non-clinical range for the duration of the study. However, this group experienced a large reduction in their time use over the 12-month period. This reduction is not necessarily problematic as this group were engaging in a very high level of activity at baseline, which then reduced to more normative levels. The exact reason for the high levels of activity at baseline requires further investigation but it could reflect over-activity resulting from mania or insomnia, which then stabilises. Alternatively, the trajectory could reflect a deteriorating profile which would be more problematic. Continuing to follow-up this group over a longer period of time would be useful in order to examine whether activity levels plateau or reduce further.

The rates of recovery outlined in this study are similar to those outlined in previous studies of FEP cohorts using alternative definitions and outcome measures. Wunderink *et al*^22^ report 26.4% of patients with FEP as functionally recovered after 2 years, with recovery defined as not experiencing any disability on any of the seven functional roles outlined in the Groningen Social Disabilities Schedule. Strakowski *et al*^23^ define recovery as 8 weeks of functioning consistently at the premorbid level, and report a 35% recovery rate after 12 months following the first admission into hospital. However, premorbid functioning may not reflect a good outcome when compared with non-clinical groups. Indeed, a strength of the current study is the use of time use as a more explicit and defined measure of social functioning which can be directly compared with non-clinical norms. Nevertheless, the current study only focuses on functional outcomes and does not take into account symptomatic recovery.

The results of the current study suggest that functional recovery from FEP may be more difficult to achieve than symptomatic recovery, with previous research indicating over 50% of patients with FEP make a symptomatic recovery.^22^ In a recent meta-analysis, rates of recovery were only 14% when both clinical and social elements were included in the definition.^24^ More targeted intervention may be required to improve social recovery from early psychosis. Part of the role of EIP is to help individuals maintain their premorbid level of functioning^25^ and the findings suggest that this occurs for some individuals. However, individuals with poor premorbid functioning may require additional assistance to improve their functional outcome.

#### Predictors of recovery

The findings of this study support previous literature identifying gender;^26^ ethnic minority status;^27^ younger age at illness onset;^28^ premorbid adjustment;^29^ and negative symptoms^30^ as predictors of poor social recovery following psychosis. It is possible that there is a common factor underlying all of these variables, influencing adaptation to psychosis and eventual social recovery. One question warranting further evaluation is the notion of social competence, referring to an individual's ability to impact favourably on their social world.^31^

Increased negative symptoms, poor adolescent premorbid adjustment, and a younger age at onset of psychosis may all reflect disruption in the development of the skills required to solve life problems and achieve instrumental and affiliative goals. Moreover, social networks have been found to be reduced in males^32^ and ethnic minorities,^33^ possibly indicating reduced social capital in these groups. This may influence the onset of psychosis but also an individual's resilience and the amount of interpersonal resources available in terms of coping with the consequences of a psychotic episode.

Interestingly, age at onset differentiated between Low Stable and High-Decreasing trajectories, with older participants more likely to be classified as high-decreasing than low stable, but not between low stable and moderate-increasing classes. This may reflect a higher baseline level of activity in individuals who are older when they develop psychosis. This is consistent with literature suggesting poorer premorbid functioning in individuals with a younger age at onset of psychosis.^28^ In addition, individuals with ethnic minority status were more likely to be classified in the Low Stable compared with the Moderate-Increasing trajectory. However, ethnicity did not differentiate between Low Stable and High-Decreasing trajectories. This could be taken to suggest that individuals with ethic minority status are less likely to experience an improvement in their functioning, consistent with literature suggesting a poorer outcome in this group.^27^ These hypotheses require further research in order for the role of age at onset and ethnicity on outcome to be better understood.

#### Social recovery *v*. symptomatic recovery

Baseline levels of positive psychotic symptoms assessed by the PANSS did not consistently predict functional outcome, although individuals in the Moderate-Increasing trajectory did have lower baseline scores on the PANSS Positive subscale. This supports literature suggesting that functional recovery can be independent from symptomatic fluctuations.^34^ This finding is also in line with patient literature outlining recovery as ‘living a meaningful life even within the constraints of mental illness’.^35^ Indeed, some individuals manage to return to a good level of functioning even if they still experience psychotic symptoms.

In addition, baseline levels of depression did not predict functional outcome. This finding contradicts previous studies highlighting increased levels of depression and low self-esteem in individuals with social recovery difficulties.^36^ However, depression levels were high in the current study, with all recovery groups scoring above the cut-off for clinical levels of depression at baseline. Such ceiling effects would make differences between the groups difficult to observe. It may be the case that it is persistent depression which predicts long-term social disability, rather than low mood observed in the early stages of psychosis. Further research is necessary to investigate this in more detail.

### Clinical implications

The results of this study suggest that certain groups of people may be more at risk of long-term social disability than others, including males and individuals from ethnic minority groups. Moreover, high levels of baseline negative symptoms and poor premorbid adjustment, specifically in late adolescence, are also indicative of social disability on entry into EIP services and poor social recovery at 12 months, as is a younger age at onset of psychosis. Individuals displaying the characteristics found to be linked with social disability may require monitoring and targeted intervention in relation to their activity levels and functional recovery.

In line with previous research, it seems that at least for some people, functional disability may occur prior to the onset of FEP. Thus, further research focusing on the prodromal phase is needed. There may be an argument for early intervention at the first stages of social disability, rather than waiting for the onset of positive psychotic symptoms.^37^ Indeed, just as research on DUP suggests that untreated psychotic symptoms may be toxic in terms of symptomatic recovery,^38^ the finding that poor premorbid adjustment predicts later social disability suggests that untreated social functioning problems may be toxic for social recovery. Interventions prior to the onset of psychosis may involve detection and monitoring of individuals displaying early signs of social disability, as well as mental health difficulties. Indeed, this supports findings that the inclusion of reductions in role functioning improves the predictive validity of at-risk mental state criteria.^39^ Engagement will also be key with this client group who may find it difficult to access services due to a high level of social exclusion.

Although, social recovery is a central feature of EIP policy and recent clinical guidelines,^25,40^ the findings of this study suggest that it may be difficult to achieve. Targeted interventions are likely to be necessary in formulating social recovery difficulties and improving functional outcomes after the onset of psychosis. Existing interventions include supported employment,^41^ cognitive remediation,^42^ cognitive–behavioural therapy for negative symptoms,^43^ and social recovery-oriented cognitive–behavioural therapy,^44^ all of which have produced promising results. Peer support groups have also been found to be useful following the onset of psychosis in order to increase social networks.^45^

### Weaknesses and future research

Although this study has highlighted several predictors of social recovery following FEP, it does not explain why or how these variables affect outcome. The study design was observational and therefore any relationship between patient characteristics is not necessarily causal. Future studies should focus on examining mediators of relationships between predictors and outcome. This will be important in identifying mechanisms of change and developing effective interventions. The current study focused on baseline predictors of outcome but did not consider factors which may contribute to recovery occurring after the onset of psychosis, such as engagement with services, the way in which individuals understood and coped with their psychotic episode, and treatment adherence. The latter is particularly relevant given the recent debate about whether lower doses of antipsychotic medication result in better outcomes.^46^ Future outcome studies should measure patterns of medication usage to investigate this further. In addition, depression was the only measure of mood in the current study, whereas anxiety is also prevalent in FEP and needs to be considered. Moreover, cognitive deficits have previously been linked with poor functional outcomes^47^ and the affect of this was not assessed in the current study.

Due to the retrospective nature of assessments of premorbid adjustment, a prospective study examining profiles of functioning using the TUS with individuals in the prodromal phases of illness would be useful in unpicking whether functional deficits highlighted in the current study were a consequence of the onset of psychosis or whether they existed premorbidly. Baseline assessments in the National EDEN study were conducted up to 3 months after being accepted into the EIP service. Thus, it was difficult to assess the impact of symptoms on functioning as the acute psychotic episode had usually been stabilised by the time of the baseline assessment.

Finally, a longer-term follow-up would enable the process of recovery from psychosis to be examined in more detail. Previous studies have examined recovery over a 2- to 7-year period.^30^ It may be the case that the first year of EIP is mostly about remission of symptoms and adjusting to the impact of the episode rather than functional recovery, which can take longer to achieve.^48^ The inclusion of additional time points would also enable non-linear models to be examined. This was not possible in the current study due to constraints of the study design meaning that only three time points were available for analysis. This is a limitation as change in social disability over time may be non-linear for some individuals. For example, functioning may fluctuate over time or plateau after an initial improvement.

This study has described the heterogeneous nature of social recovery in FEP using weekly hours in structured activity as an index of social functioning. The findings suggest a large proportion of individuals remain socially disabled after 12 months of EIP service provision. Predictors of social recovery were identified, suggesting that males and individuals from ethnic minority groups may be at risk of social disability following FEP. In addition, individuals with a young age at onset of psychosis, high baseline levels of negative symptoms, and poor adolescent premorbid adjustment may also be at risk. Individuals with one or more of these indicators may require close monitoring and targeted intervention in relation to improving their social outcome following FEP. Future research should focus on developing understanding of the way in which predictors affect outcome, thus identifying potential mechanisms of change to inform intervention development. Moreover, a longer period of follow-up is required to examine social recovery over the full duration of EIP and beyond. Examining changes in functioning during the premorbid and prodromal phases will also be important in understanding when social disability becomes a problem and identifying potential windows for intervention.
